# Total laparoscopic versus laparoscopic-assisted transabdominal posterior mediastinal digestive tract reconstruction in the treatment of Siewert II adenocarcinoma of the esophagogastric junction: A retrospective study

**DOI:** 10.3389/fsurg.2022.874857

**Published:** 2022-08-19

**Authors:** Liang Wang, Xiaoqian Chen, Wei Miao, Yubin Ma, Xinfu Ma, Chun Wang, Xiaobo Cao, Hongyin Xu, Jiajia Wei, Su Yan

**Affiliations:** Department of Gastrointestinal Oncology, Qinghai University Affiliated Hospital, Xining, China

**Keywords:** total laparoscopy, posterior mediastinum, Siewert type II, adenocarcinoma of the esophagogastric junction, digestive tract reconstruction

## Abstract

**Background:**

The method of operation and the range of resection for Siewert II adenocarcinoma of the esophagogastric junction (AEG) remain controversial. This study aims to evaluate the safety, feasibility, and short-term postoperative effect of total laparoscopic versus laparoscopic-assisted transabdominal posterior mediastinal digestive tract reconstruction in the treatment of Siewert II AEG.

**Methods:**

Total laparoscopic or laparoscopic-assisted gastrointestinal reconstruction through abdominal posterior mediastinum was performed in 108 patients with Siewert II AEG from October 2017 to February 2019. This study evaluated the loss of intraoperative blood, the number of lymph nodes, the marginal of the tumor, short-term postoperative complications (within 30 days), the rate of survival at follow-up, and the economic cost, feasibility, and effect of short-term postoperative recovery for patients who received these two operations.

**Result:**

There were no significant differences in general data between the total laparoscopic group and the laparoscopic-assisted group (*P > *0.05). However, the total laparoscopic group cost more time on the surgical procedure and digestive tract reconstruction, lost less intraoperative blood, and had more mediastinal lymph nodes compared with the laparoscopic-assisted group (*P < *0.05). The total laparoscopic group was significantly better than the laparoscopic-assisted group compared with the short-term postoperative recovery indexes, such as the first exhaust time, the first defecation time, the first fluid time, the first semi-fluid diet time, the postoperative hospital stay, and other postoperative recovery indexes (*P < *0.05). In addition, there were no significant differences in postoperative complications, postoperative pathological indexes, the recurrence rate, and mortality between the total laparoscopic group and laparoscopic-assisted group (*P > 0.05*).

**Conclusions:**

The safety, feasibility, and short-term effect of total laparoscopic transabdominal posterior mediastinal digestive tract reconstruction in the treatment of Siewert II AEG were better than those for the laparoscopic-assisted group.

## Introduction

Adenocarcinoma of the esophagogastric junction (AEG) refers to the adenocarcinoma that occurs within the range of 5 cm above and below the anatomic boundary of the esophagus and stomach. AGE is classified as an independent disease because of the particularity of its anatomical location and biological behavior (1). The standard of AEG classification recognized by researchers is proposed by Professor Siewert of the University of Munich in Germany (2): the tumor is 1–5 cm above the dentate line (type I), the tumor is located from 1 cm of the supra dentate line to 2 cm of the borderline (type II), and the tumor is 2–5 cm below the dentate line (type III). The incidence of three Siewert types of AEG is similar in European countries, but Siewert II and III are mainly found in Asian countries (3, 4). Studies have shown that the incidence of AEG is still increasing, and most of the patients with AGE are in the progressive stage (5, 6). In addition, the prognosis of AGE is poor, and the 5-year survival rate of AGE is less than 30% because of its special biological behavior (7). The treatment of AEG is regarded as a challenging treatment problem that has aroused widespread concern among clinicians (8). At present, there are many disputes about the tumor node metastasis (TNM) stage, the choice of surgical approach, the scope of lymph node dissection, digestive tract reconstruction, and perioperative adjuvant therapy for AGE (9–11). In particular, the transthoracic approach and transabdominal retromediastinal approach, as the two common surgical approaches in AEG, have their own advantages and disadvantages. Furthermore, there is no other solution to solve the above problems comprehensively (12). It is an important concern to find an optimized treatment strategy for AEG.

Laparoscopic surgery has been widely used in treating gastric cancer since Kitano et al. first reported the application of laparoscopic-assisted radical resection of distal gastric cancer in 1994. Previous studies have confirmed the safety and effectiveness of laparoscopic surgery (13–16). Nowadays, with the development of endoscopic technology, laparoscopic magnification technology can show the fine structure of the vascular system, nerve, and fascia, which makes this method of operation to have a special advantage in clearly identifying each anatomical level during the operation.

In recent decades, total gastrectomy or proximal gastrectomy can be selected for patients with AEG according to the invasion degree and lymph node metastasis of tumors (17, 18). Many surgeons have rich experience in surgical techniques for laparoscopic-assisted radical gastrectomy. In contrast, the number of total laparoscopic radical gastrectomies is limited by technical challenges and a lack of long-term research results. In recent years, many scholars have reported the study of total laparoscopic radical resection for distal gastric cancer and used the intraluminal digestive tract reconstruction technique. Compared with laparoscopic-assisted radical resection, total laparoscopic radical resection for distal gastric cancer caused smaller incisions and less invasion, especially in obese patients. The approach of total laparoscopic can reduce the postoperative incision fat liquefaction and the risk of incision infection in obese patients. Total laparoscopic reconstruction of the digestive tract *in vivo* has become the first choice for early gastric cancer because of its advantages, such as no retraction of incision, wide field of vision, and tension-free anastomosis. However, most surgeons prefer laparoscopic-assisted digestive tract reconstruction to total laparoscopic digestive tract reconstruction because total laparoscopic digestive tract reconstruction has difficulties in intraluminal digestive tract reconstruction, especially when performing laparoscopic esophageal–jejunal digestive tract reconstruction (19). There is an urgent need to overcome the technical difficulties of total laparoscopic surgery and apply it to the treatment of AEG.

## Materials and methods

### Study population

A total of 532 upper gastric cancer patients undergoing surgery in the affiliated Hospital of Qinghai University from October 2017 to February 2019 were included. The percentage of total laparoscopy was 40.7%. The rates of total gastrectomy, proximal gastrectomy, and other anastomotic types accounted for 47.59%, 28.28%, and 24.13%, respectively. In this current study, a table of random digits was used to select 64 patients with Siewert II AEG who were treated with laparoscopy-assisted technology, and 44 patients were received total laparoscopic gastrointestinal reconstruction technology. Finally, a total of 108 patients with Siewert II AEG who underwent laparoscopic digestive tract reconstruction through abdominal posterior mediastinum were analyzed retrospectively. The general information of the patients is shown in Table 1.

**Table 1:** Preoperative patient demographic information.

	Laparoscopy-assisted group (*n* = 64)	Totally endoscopic Group (*n* = 44)	*P* value
Age (years)	62.01 ± 8.31	59.72 ± 8.64	0.170
Gender			
Male	50 (78.13%)	38 (86.36%)	0.279
Female	14 (21.87%)	6 (13.64%)	
BMI (Kg/m^2^)	21.97 (20.33–22.96)	22.08 (21.14–23.18)	0.058
ASA grade			
I	35 (54.68%)	31 (70.45%)	0.194
II	22 (34.37%)	7 (15.91%)	
III	7 (10.93%)	6 (13.63%)	
Preoperative CEA (ng/L)	2.44 (1.55–10.80)	2.12 (1.45–4.96)	0.348
preoperative examination time (d)	9 (7-10)	9 (7-10)	0.947
Previous surgical history			
No	38 (59.37%)	33 (86.40%)	0.093
Yes	26 (40.62%)	11 (13.60%)
Previous abdominal surgical history			
No	53 (82.81%)	38 (86.36%)	0.083
Yes	11 (17.19%)	6 (13.64%)	
Basic diseases			
No	42 (65.62%)	32 (72.72%)	
Hypertension	8 (12.50%)	2 (4.54%)	
Diabetes	4 (6.25%)	4 (9.09%)	0.663
Chronic bronchitis	6 (9.37%)	4 (9.09%)	
Heart disease	4 (6.25%)	2 (4.54%)	

BMI, body mass index; ASA , American Society of Anesthesiologists; CEA, carcinoembryonic antigen

The study was approved by the Ethics Committee of the Qinghai University Affiliated Hospital, and all patients signed informed consent forms (approval number: P-SL-20190003).

### Preoperative evaluation and surgical procedure

The inclusion criteria of the study patients were as follows: (1) the diagnosis of Siewert II gastroesophageal junction adenocarcinoma was confirmed by ultrasound gastroscopic pathological biopsy before operation; and (2) the preoperative T staging ranges from T1 to T3. Exclusion criteria are as follows: (1) Siewert type I and III patients; (2) patients with severe heart, lung, and other important organ diseases who could not tolerate operation or patients who have other surgical contraindications; (3) patients with secondary metastasis of tumor; and (4) patients without complete clinical medical records.

Patients were divided into the total laparoscopic group or laparoscopic-assisted group using a table of random digits. Both groups of operations were performed by the same experienced endoscopic surgeon, and the surgical endoscopic equipment for both groups were Olympus OVT-S190. In addition, the energy device used for surgical operations was an ultrasonic activation device. Gastrointestinal amputations were performed with a disposable linear cutting occluder in both groups. However, circular or linear staplers were used for gastrointestinal reconstruction. We will complete immune checkpoint monitoring for patients with positive proximal and distal margins. Then, SOX and immune checkpoint inhibitors will be used for combination chemotherapy.

**Figure 1:**
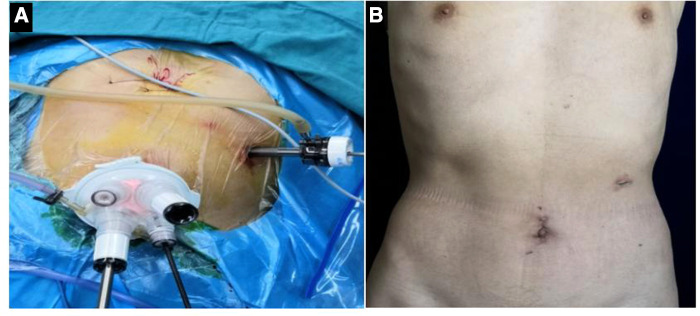
(**A**) Location of intraoperative incision; (**B**) postoperative abdominal incision was performed.

1. After successful anesthesia, the patient lay on his back with his legs apart, in a “human” shape, keeping the head high and the feet low. The operation routine included iodophor disinfection and towel laying.
2. An arc skin incision of about 4 cm was made in the umbilicus, and an incision in the abdominal cavity at each layer was made. The incision was placed in a card seat after placing a folding clip and connected with a pneumoperitoneum tube to make a pneumoperitoneum. An indwelling 12-mm indent was placed 2 cm from the left border of the anterior axillary costal space as butcher's hole operation (Figures 1A,B). In the laparoscopic-assisted group, after the completion of endoscopic dissociation, a longitudinal incision of about 10 cm was made under the median xiphoid process of the upper abdomen, entered the abdomen layer by layer, and underwent gastrointestinal anastomosis.

### Total laparoscopic abdominal and mediastinum after esophagus jejunum Roux-en-Y anastomosis

After the mediastinal lymph nodes were completely removed, the esophagus was severed with a linear cutting stapler at a distance of 3 cm from the tumor edge, and the end of the esophagus was seen retracting into the mediastinum and thoracic cavity. At this point, the position was adjusted to 30° above the head and tilted 30° to the right. The distal end of the Y-arm of the jejunum entered the hiatus. If the space was narrow, a part of the diaphragm was removed at 1.5 cm from the diaphragm of the left foot (usually, the length of the cut was 3–4 cm). We even needed to enter from the left side of the chest to see the left side of the lower lung.

**Figure 2:**
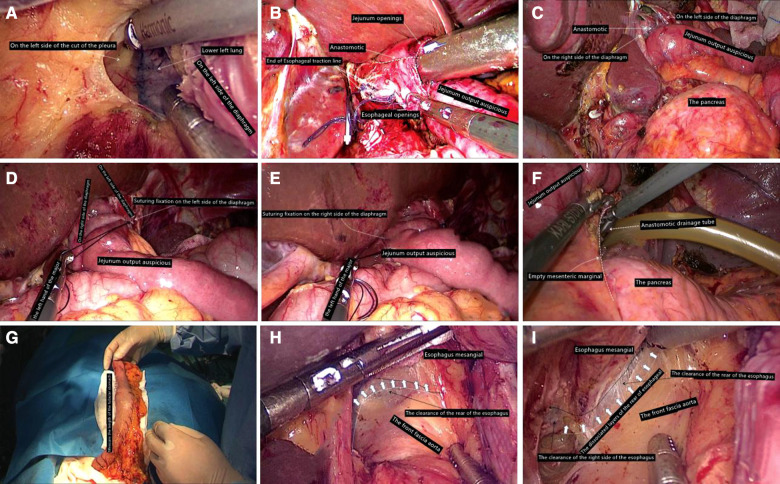
(**A**) Enter the left chest; (**B**) leave the esophageal stump to use traction line for esophagus jejunum anastomosis; (**C**) inspire stapling anastomosis. (**D**) On the left side of the diaphragm foot, suture fixation is completed. (**E**) Diaphragm on the right foot with jejunum output auspicious suture fixation has been completed. (**F**) Through the left rear of esophageal hiatus, put drainage tube into mediastinum anastomosis. (**G**) Side of the stomach tube toward the head direction of traction, perform the stomach tube length measurement to ensure that the tubular stomach without any tension. (**H**) Ensure mesangial esophagus and aorta front fascia surface's integrity. (I) Go through the right side of the esophageal clearance and pay attention not to damage the mediastinal pleura as far as possible.

The purpose was to ensure the operation in an ideal space and avoid the inability of the Y-arm jejunum and small mesentery to enter the mediastinum, which will bring difficulties to the next matching. On the right side of the esophagus, we used a 3-0 gastrointestinal to suture two preset traction lines with a length of about 6 cm and then, under the guidance of the catheter, used an ultrasonic knife to cut a hole with a diameter of 0.5 cm at the end of the catheter. On the left side of the edge, a line was drawn on top of the full thickness suture needle to complete the preparation of the tube side. Then, a hole of the same diameter was punched from the tip of the Y-arm of the jejunum to 4.5 cm from the contralateral mesentery, and each layer of the predrawn wire was sutured. The thick end of the linear cutting stapler was placed in the small intestine. The thin end (to the side of the nail seat) was placed in the esophagus opening, and the preset wire was pulled out in the opposite direction of moderate traction of the stapler. It must be carefully checked whether the anastomosis between the esophageal mucosa and the jejunal mucosa was complete, whether there was bleeding at the anastomosis between the esophageal mucosa and the jejunal mucosa, whether the outside of the anastomosis was complete, and whether there were accidents such as perforation (Figures 2A–C). The opening of the esophagus and jejunum was usually closed with a combined opening suture (or ordinary 3-0 gastrointestinal suture) to complete continuous full-thickness suture closure with double suture reinforcement. After the entire procedure was complete, the area around the anastomosis must be examined. If there was a problem, especially the top anastomotic, it was repaired with a needle if necessary and the number of suture stitches was strengthened intermittently. In order to prevent anastomotic regurgitation, we recommended hiatus reconstruction. The output part of the jejunum was usually fixed with a needled suture around the diaphragmatic foot (Figures 2D,E). Finally, two drainage tubes were usually placed in the mediastinum after the left and right anastomosis (Figure 2F).

### Total laparoscopic abdominal and mediastinal type tube after gastric and esophageal carcinoma

Proximal gastrectomy can be performed for Siewert type II AEG primary cancer with a lesion less than 4 cm in diameter. The distal end of the stomach was straightened along to complete the tubular gastric anastomosis. We believe that the diameter of the tubular stomach is 3–4 cm, and attention should be paid to ensuring the integrity of the vascular arch on the greater curvature and the tubular stomach. It is required to remove part of the stomach fundus and insert reinforcement, and the final tubular stomach is about 3 cm in diameter. Two sutures were fixed at the preset end of the esophagus and stomach. Then, we need to change and cover the patient's position. The esophagus was completely freed and excised under thoracoscopy. The gastric tube was inserted into the thoracic cavity to avoid the inversion of the gastric tube. The side of the gastric tube and the esophagus were punched, and a 45-mm straight stapler was used to complete the complete overlapping anastomosis of the gastric tube and the esophagus. After the anastomosis was completed, the tubular stomach was fixed to the hiatus area (Figure 2G).

### Statistical analysis

SPSS 25.0 was used to analyze the study data. The measurement data conformed to a normal distribution will be expressed by the mean and standard deviation (*X ± S*) and compared using the *t* or *t^/^* test of two independent samples. The measurement data that did not conform to a normal distribution will be expressed by *M* (*Q_L_−Q_U_*) and compared using the rank sum test. The qualitative data were tested by a *X*^2^ test or rank sum test. When the test *P *<* *0.05, the difference was statistically significant. GraphPad Prism 7.00 software was used for graphic statistics.

## Results

### General data

A total of 108 patients with Siewert type II AEG who underwent gastrointestinal reconstruction through the retroabdominal mediastinum were divided into the laparoscopic-assisted group (*n* = 64) and the total laparoscopy group (*n* = 44). There were 64 patients in the laparoscopic-assisted group, including 50 males (78.12%) and 14 females (21.87%), with an average age of 62.01 years. In addition, a total of 44 patients were included in the total laparoscopy group, including 38 males (86.36%) and 6 females (13.63%), with an average age of 59.72 years. Statistical analysis showed that there were no significant differences in sex ratio and age between the two groups. Moreover, there were no significant differences between the two groups in terms of body mass index (BMI), previous surgical history, previous abdominal surgical history, and basic diseases (see Table 1).

### Total laparoscopy group takes longer, but the amount of blood loss is low and the number of mediastinal lymph nodes is more

The postoperative results showed that in the laparoscopic-assisted group, total gastrectomy was performed in 28 cases (43.75%) and proximal gastrectomy was performed in 36 cases (56.25%). One patient was converted to laparotomy because of intraoperative common hepatic artery injury and poor laparoscopic hemostasis. In the total laparoscopic group, 18 patients (40.91%) underwent total gastrectomy, 26 patients (59.09%) underwent proximal gastrectomy, and no patients were transferred to exploratory laparotomy. Mime et al. (20) analyzed 288 Siwert type II AEG cases with the R0 resection in seven Japanese seven centers. Its results found that when the tumor was resected ≤30 mm from the dentate line, the incidence of lymph node metastasis in the greater curvature and the superior and inferior pylorus was <2.2%, and when the tumor was >50 mm away from the dentate line, the lymph node rate reached 20.0%. Therefore, the plan for the scope of gastrectomy can be based on the distance between the distal end of the tumor and the dentate line. Proximal gastrectomy was suggested when the distance between the tumor and the dentate line was ≤30 mm, and total gastrectomy was recommended when the distance was >50 mm.

**Figure 3:**
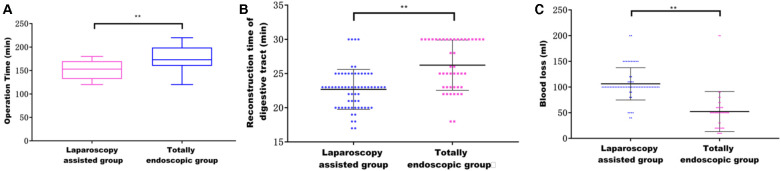
(**A**) Effect of different surgical methods on operation time; (**B**) effect of different surgical methods on the time of digestive tract reconstruction; (**C**) effect of different surgical methods on intraoperative blood loss.

**Figure 4:**
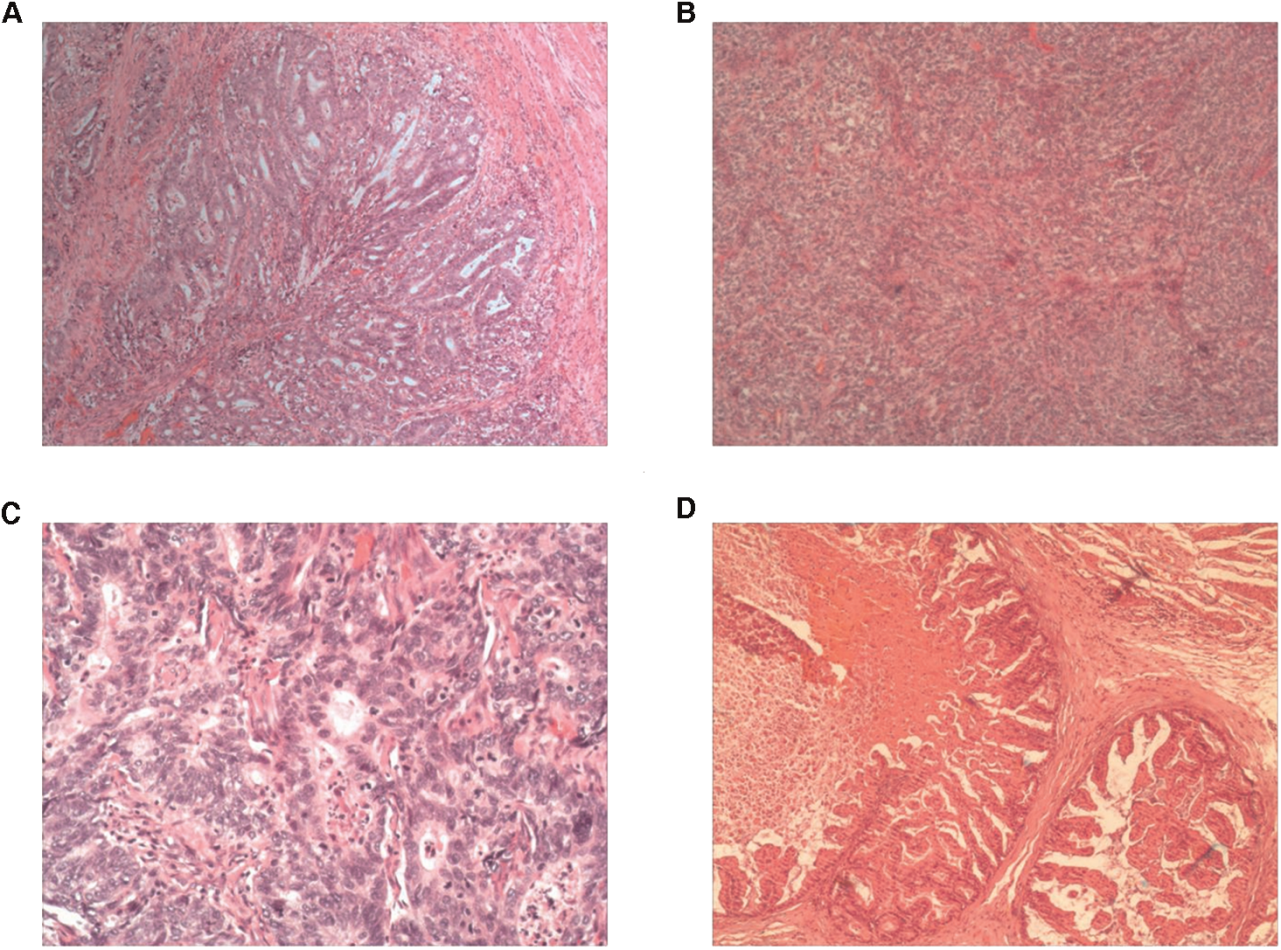
(**A**) Pathology results from the totally endoscopic group by total gastrectomy; (**B**) pathology results from the laparoscopy-assisted group by total gastrectomy; (**C**) pathology results from the totally endoscopic group by proximal gastrectomy; (**D**) pathology results from the laparoscopy-assisted group by proximal gastrectomy.

Both groups underwent total or proximal gastrectomy in the current study. Our results showed that a higher proportion of patients in the laparoscopic-assisted group underwent total gastrectomy. However, there was no statistical difference in surgical modality between the two groups. The operation time and digestive tract reconstruction time of the total laparoscopy group were longer than those in the laparoscopy-assisted group (*P* < 0.05) (Figures 3A,B). This may be due to the fact that total laparoscopic surgery is more difficult than laparoscopic-assisted surgery and requires highly skilled surgeons, especially in the step of endoscopic gastrointestinal reconstruction. It was also found that intraoperative blood loss was lower in the total laparoscopic group than that in the laparoscopic-assisted group (*P *<* *0.05) (Figure 3C). However, tumor parameters were not significantly different between the two groups (*P *>* *0.05). The postoperative pathological report showed that there was no significant difference in the number of abdominal lymph nodes between the two groups (Figures 4A–D), indicating that total laparoscopic surgery and laparoscopic surgery implemented the same principle of radical resection and safety of tumor. More mediastinal lymph nodes were obtained in the total laparoscopic group than in the laparoscopic-assisted group.

The prognosis of patients with Siewert II AEG with predominantly abdominal lymph node metastases that invade the lower esophagus leading to mediastinal lymph node metastases is very important (see Table 2). Compared with the laparoscopic-assisted group, total laparoscopic retroperitoneal mediastinal gastrointestinal reconstruction cost more time, but the blood loss was low, the numbers of removed mediastinal lymph nodes were more, and the tumor resection effect was better.

**Table 2:** intraoperative correlation index.

	Laparoscopy assisted group (*n* = 64)	Totally endoscopic group (*n* = 44)	*P* value
Surgical procedure			
TG	28 (43.75%)	18 (40.91%)	0.769
PG	36 (56.25%)	26 (59.09%)	
Operation Time (min)	153.00 (132.00–170.00)	173.00 (160.00–199.00)	**0.000**
Reconstruction time of the digestive tract (min)	23.00 (20.00–25.00)	25.50 (23.00–30.00)	**0.000**
Blood loss (ml)	100.00 (100.00–110.00)	50.00 (20.00–60.00)	**0.000**
Surface area of serosa (cm)	0.00 (0.00–0.95)	0.00 (0.00–2.00)	0.188
Near cutting edge (cm)	3.00 (3.00–3.00)	3.00 (2.50–3.00)	0.336
Distance between tumor distal end and EGJ line (cm)	3.00 (2.65–5.37)	3.00 (2.35–5.27)	0.259
Number of mediastinal lymphadenectomy	0.00 (0.00–0.00)	0.00 (0.00–2.00)	**0.000**
Number of positive mediastinal lymph nodes	0.00 (0.00–0.00)	0.00 (0.00–0.00)	0.087
Number of abdominal lymph node dissections	23.00 (18.00–30.00)	26.00 (22.00–30.00)	0.160
Positive number of abdominal lymph nodes	1.00 (0.00–5.00)	1.00 (0.00–3.00)	0.723

PG, proximal gastrectomy; TG, total gastrectomy; min,minute; ml,milliliter; cm, centimetre. Bold values indicate, when the test *P* < 0.05, the difference was statistically significant.

### Total laparoscopic surgery can significantly improve postoperative pain and accelerate postoperative recovery

**Figure 5:**
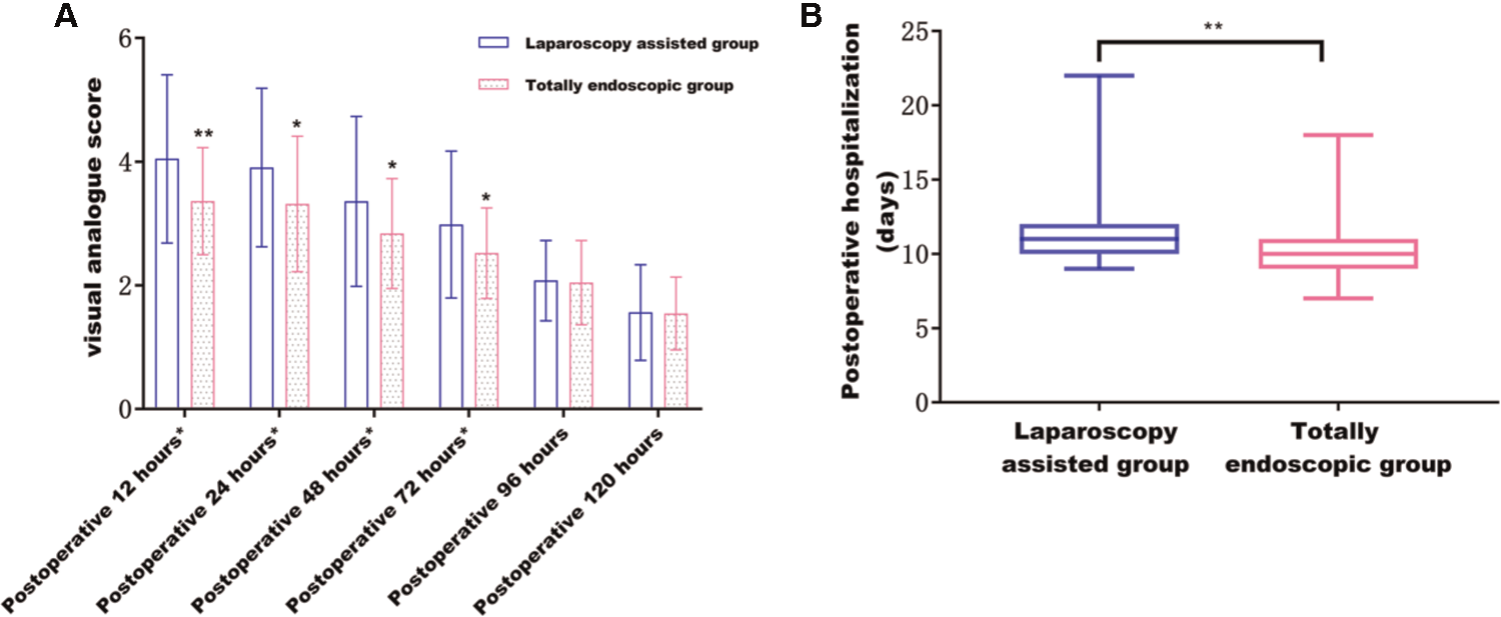
(**A**) Effect of different surgical methods on postoperative pain from 12 to 120 h; (**B**) effect of different surgical methods on postoperative hospital stay.

The postoperative pain in the total laparoscopic group was not only significantly improved within 3 days after the operation (according to the VAS score) (Figure 5A) but also superior to the laparoscopic-assisted group in the postoperative recovery index from the perspective of postoperative recovery. For example, time to the first flatulence, time to the first defecation, time to the first fluid, time to the first semi-fluid diet, the postoperative hospital stay (Figure 5B), and the total hospital stay were all shorter in the total laparoscopic group than in the laparoscopic-assisted group (*P *< 0.05). These results showed that total laparoscopic surgery could significantly improve postoperative pain and accelerate postoperative recovery compared with laparoscopic-assisted surgery (see Tables 3 and 4).

**Table 3:** postoperative recovery index.

	Laparoscopy-assisted group (*n* = 64)	Totally endoscopic group (*n* = 44)	*P* value
Postoperative CA19-9 (U/ml)	5.94 (2.82–11.50)	5.56 (3.26–9.56)	0.599
Postoperative CEA (ng/L)	1.73 (1.26–4.99)	2.44 (1.30–3.44)	0.468
The first time to get out of bed after operation (days)	1.56 ± 0.65	1.27 ± 0.45	0.070
Postoperative exhaust time (days)	3.00 (3.00–4.00)	3.00 (2.00–3.00)	**0.000**
Postoperative defecation time (days)	4.00 (3.00–4.00)	3.00 (3.00–3.00)	**0.002**
Time of fluid diet after the operation (days)	10.00 (9.00–12.00)	9.00 (9.00–11.00)	**0.002**
Postoperative half-fluid diet time (days)	13.00 (12.25–14.00)	12.00 (11.00–13.75)	**0.001**
Postoperative hospitalization (days)	11.00 (10.00–12.00)	10.00 (9.00–11.00)	**0.001**
Length of postoperative hospital stay (days)	11.00 (10.00–12.00)	10.00 (9.00–11.00)	**0.001**
Operation expenses (CNY)	27650.00 (20680.00–32928.50)	29086.00 (26272.00–33738.25)	0.087
Total hospitalization expenses (CNY)	51061.51 (47898.46–58303.01)	52771.10 (48762.13–70265.04)	0.310

CA19-9,carbohydrate antigen 19-9; CEA, carcinoembryonic antigen; d,day; CNY,Chinese Yuan. Bold values indicate, when the test *P* < 0.05, the difference was statistically significant.

**Table 4:** postoperative recovery index.

Postoperative VAS score	Laparoscopy-assisted group (*n* = 64)	Totally endoscopic group (*n* = 44)	*P* value
VAS score 12 h after the operation	4.04 ± 1.36	3.36 ± 0.86	**0.002**
VAS score 24 h after the operation	3.90 ± 1.28	3.31 ± 1.094	**0.015**
VAS score 48 h after the operation	3.35 ± 1.37	2.84 ± 0.88	**0.019**
VAS score 72 h after the operation	2.98 ± 1.18	2.52 ± 0.73	**0.024**
VAS score 96 h after the operation	2.07 ± 0.64	2.04 ± 0.68	0.802
VAS score 120 h after the operation	1.56 ± 0.77	1.54 ± 0.58	0.902

VAS,visual analogue score. Bold values indicate, when the test *P* < 0.05, the difference was statistically significant.

### Total laparoscopic surgery is safe and effective

**Figure 6:**
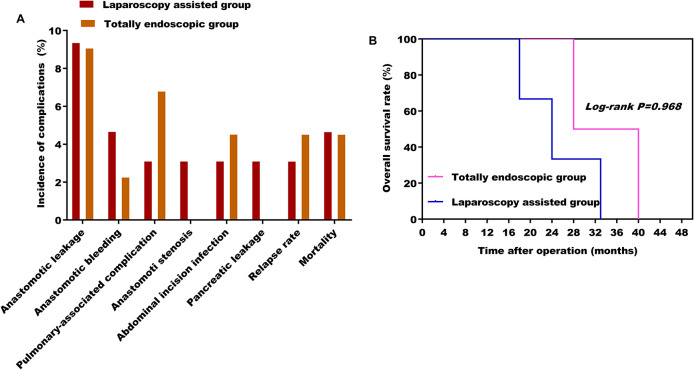
(**A**) Effect of different surgical methods on the incidence of postoperative complications; (**B)** effect of different surgical methods on overall survival rate.

According to the Clavien–Dindo complication grading standard, all patients with complications in this study were ≤III^a^. There were no significant differences in postoperative complications, proximal and distal incisal marginal status, recurrence rate, and mortality rate (Figure 6A) between the two groups (* P*> 0.05). A total of four patients recurred. Of these, two cases in the laparoscopic-assisted group underwent total gastrectomy for secondary liver malignant tumors at 16 and 28 months after the operation. In the total laparoscopic group, there were two cases, of which one case underwent total gastrectomy for secondary liver malignancy 21 months after the operation and one case underwent proximal gastrectomy for secondary peritoneal malignancy 30 months after the operation. No death occurred in both groups during hospitalization. However, two patients in the laparoscopic-assisted group died of liver failure caused by secondary malignant liver tumor within 24 months and 33 months after total gastrectomy. One patient died unexpectedly because of a cerebral hemorrhage 18 months after proximal gastrectomy. Two patients in the total laparoscopic group recurred and died of liver failure because of secondary malignant liver tumors at 28 months after total gastrectomy and 40 months after proximal gastrectomy, respectively (Figure 6B) (see Tables 5 and 6). In general, total laparoscopic surgery has a higher safety and better resection effect than laparoscopic-assisted surgery.

**Table 5:** postoperative pathological indexes.

	Laparoscopy-assisted group (*n* = 64)	Totally endoscopic group (*n* = 44)	*P* value
Postoperative T staging			
T_1_	9 (14.06%)	10 (22.73%)	0.446
T_2_	29(45.31%)	16 (36.36%)	
T_3_	26 (40.63%)	18 (40.91%)	
Postoperative N staging			
N_0_	34 (53.13%)	19 (43.18%)	0.579
N_1_	21 (32.81%)	20 (45.45%)	
N_2_	8 (12.50%)	4 (9.09%)	
N_3_	1 (1.56%)	1 (2.28%)	
Differentiated degree
Poorly differentiated	30 (46.88%)	26 (59.09%)	
Moderately differentiated	26 (40.62%)	14 (31.82%)	0.456
High differentiated	8 (12.50%)	4 (9.09%)	
Near margin status			
Positive	1 (1.56%)	0 (0%)	0.405
Negative	63 (98.44%)	44 (100%)	
Distal margin status			
Positive	0 (0%)	1 (2.27%)	0.226
Negative	64 (100%)	43 (97.73%)	

**Table 6:** postoperative safety index.

	Laparoscopy-assisted group (*n* = 64)	Totally endoscopic Group (*n* = 44)	*P* value
Morbidity < grade III*	17 (26.56%)	10 (22.72%)	0.651
Anastomotic leakage	6 (9.37%)	4 (9.09%)	0.960
Anastomotic bleeding	3 (4.68%)	1 (2.27%)	0.514
Pulmonary-associated complication	2 (3.12%)	3 (6.81%)	0.369
Anastomoti stenosis	2 (3.12%)	0 (0%)	0.237
Abdominal incision infection	2 (3.12%)	2 (4.54%)	0.701
Pancreatic leakage	2 (3.12%)	0 (0%)	0.237
Relapse rate	2 (3.12%)	2 (4.54%)	0.701
Mortality	3 (4.68%)	2 (4.54%)	0.972

*According to the Clavien-Dindo grading system.

## Discussion

At present, scholars at home and abroad have reached a certain consensus on the surgical path, surgical resection range, and lymph node dissection range of Siewert type I and III AEG. However, due to the particularity of the anatomical position of Siewert II AEG, its tumor biological behavior is complex, which brings some difficulties to its treatment. The main treatment method of Siewert II AEG is a surgery-based comprehensive treatment. According to the tumor invasion and lymph node metastasis, total gastrectomy or proximal gastrectomy will be selected. This study discussed the advantages and disadvantages of laparoscopic-assisted and total laparoscopic transabdominal posterior mediastinal gastrointestinal reconstruction in the treatment of Siewert II AEG and laid a foundation for the further application of total laparoscopy in the clinical treatment of Siewert II AEG.

Among the Siewert II AEG patients included in this study, the proportion of male patients was higher than that of female patients. This result was basically similar to that reported in other foreign studies (21, 22). However, unlike what was reported by Hosokawa (23), there were no significant differences in the gender distribution of the three types of AEG in this study. We consider that this difference may be closely related to factors such as sample size and ethnicity. The epidemiological gender distribution of Siewert II AEG deserves further study. The results of BMI data showed that although there was no significant difference in BMI between the two groups, the average BMI in the total laparoscopy group was higher than that in the laparoscopic-assisted group and the number of patients with BMI greater than 25 kg/m^2^ in the total laparoscopic group was more than that in the laparoscopic-assisted group. BMI with higher values was more likely to lead to postoperative complications, such as incision fat liquefaction and incision infection, thus aggravating the postoperative pain and prolonging the hospitalization time of the patients. Therefore, it is speculated that obesity may be a risk factor for poor results of early surgery. The operation time in the total laparoscopic group was found to be longer than that in the laparoscopic-assisted group, which was similar to the average operation time of 183 min reported in other studies (24, 25). The reason may be that total laparoscopic surgery is difficult to perform and requires highly skilled surgeons, especially when reconstructing the digestive tract by endoluminal esophagojejunostomy. The exposed esophageal stump retracts easily into the thoracic cavity (26). A wide surgical field of vision and highly skilled doctors during surgery can ensure the safety of digestive tract reconstruction as much as possible and avoid postoperative anastomotic leakage. However, the intraoperative blood loss in the total laparoscopic group was significantly low than that in the laparoscopic-assisted group, which was related to the small incision, less invasion, and experience level of the surgeons. These results suggest that total laparoscopic transabdominal retromediastinal digestive tract reconstruction for patients with Siewert type II AEG has low intraoperative blood loss and less damage to the patients themselves. However, total laparoscopic surgery will take more time and require more experienced and skilled doctors.

Groups 1, 2, 3, 7, 8, and 110 were the main types of lymph node metastasis in Siewert II AEG, followed by groups 11, 111, and 9. The main way of lymph node metastasis in Siewert II AEG was abdominal lymph node metastasis. The extent of mediastinal lymph node dissection depends on the tumor size, the distance to invade the lower esophagus, tumor differentiation, and clinical stage. Whether there is abdominal and mediastinal lymph node metastasis in Siewert II AEG and whether the operator can thoroughly dissect the lymph nodes that may have metastasized during operation have an important impact on the survival and prognosis of the patient. In addition, it is extremely important to thoroughly remove the primary tumor and dissect lymph nodes during operation. It was found that there was no significant difference in the number of abdominal lymph nodes between the two groups by comparing the laparoscopic-assisted group and the total laparoscopic group (*P* > 0.05), indicating that total laparoscopic surgery can achieve the same radical tumor resection principle as the laparoscopic-assisted surgery. It was also found that there was no significant difference in the positive rate of a proximal margin between the two groups (*P* > 0.05). However, the postoperative pathological reports of both groups showed positive cases of surgical margin, which may be related to the uncertainty of the results of intraoperative rapid pathological freezing. Sufficient scope of tumor resection and lymph node dissection is still the main factor in laparoscopic surgery for malignant tumors. Especially for the advanced AEG, laparoscopic surgery can remove enough lymph nodes and obtain enough tumor margin and follow the principle of the radical tumor. Although extensive resection and lymph node dissection can obtain sufficient margins to avoid residual tumor cells and obtain more lymph nodes, this process may lead to a higher rate of postoperative complications and mortality. Therefore, extended resection is not necessary. None of the patients in this study underwent extended resection. It can be seen that efforts to enhance the true sense of R0 resection and adequate lymph node dissection are important means to improve the long-term survival rate of AEG patients. In addition, it is also the goal of digestive tract reconstruction through abdominal retromediastinum for Siewert II AEG in the total laparoscopy group.

There was no death in the two groups during the perioperative period. No statistical differences were found in the preoperative examination time and surgical methods between the two groups (*P* > 0.05). However, the postoperative hospital stay in the total laparoscopic group was shorter than that in the laparoscopic-assisted group (*P* < 0.05). This may be because compared with the laparoscopic-assisted group, the total laparoscopic group can accelerate postoperative recovery, reduce postoperative pain, and shorten postoperative hospital stay. In addition, there was no significant difference in the cost of operation and hospitalization between the two groups (*P* > 0.05). These results showed that the total laparoscopic group did not increase the economic burden on the patients. Total laparoscopic surgery may be an economical and feasible treatment for patients with AGE. Moreover, there was no significant difference in the recurrence rate and mortality rate of early postoperative complications and short-term follow-up between the two groups (*P* > 0.05). This result also suggested that total laparoscopic surgery tends to be more technically difficult than laparoscopic-assisted surgery and has a higher failure rate of intraoperative gastrointestinal reconstruction. However, the total laparoscopic surgery in this study remains safe and effective for patients. At present, there are few retrospective studies on the safety, feasibility, and short-term postoperative efficacy of total laparoscopy and laparoscopic-assisted transabdominal retromediastinal gastrointestinal reconstruction in the treatment of Siewert II AEG. We conducted this study, and we achieved better results compared to the predecessors to further explain the differences.

It was doubted whether other factors would affect the occurrence of complications in this study in evaluating the differences in the outcome of complications between the two groups. We studied and analyzed some factors that might lead to complications. First, we divided the whole cohort into two groups, including 81 patients without complications and 27 patients with complications, and the Clavien–Dindo grade of complications between the two groups was ≤IIIa. The results of this study showed that the proportion of postoperative complications in male patients was higher than that in female patients. The reason for this difference may be the higher proportion of male patients in the study by Hosokawa et al. However, the gender has no significant effect on the occurrence of complications in this study. However, the study found that age, BMI, tumor size, operation time, and intraoperative blood loss did not significantly affect the incidence of complications in this study. Our data showed that the higher the incidence of postoperative complications, the older the age, the higher the BMI value, the larger the tumor volume, the longer the operation time, and the greater the intraoperative blood loss.

In addition, it was found that the surgical method and the extent of surgical resection had no significant effect on the occurrence of complications. This result suggested that total laparoscopic surgery has no obvious disadvantage compared with laparoscopic-assisted surgery, and it is expected to be better used in clinical treatment (see Table 7).

**Table 7:** Clinicopathological factors in patients with or without morbidity ≤ grade III^a^.

	Morbidity (–) (*n* = 81)	Morbidity (+) (*n* = 27)	*P* value
Age (years)	61.00 (54.00–67.00)	63.00 (60.00–65.00)	0.757
Sex			0.055
Male	76 (93.83%)	22 (81.48%)	
Female	5 (6.17%)	5 (18.52%)	
Body mass index (Kg/m^2^)	22.04 (20.52–24.93)	21.63 (20.39–23.00)	0.081
Tumor size (cm)	5.60 (4.15–7.15)	6.00 (4.00–8.40)	0.168
Laparoscopy assisted group	45 (55.56%)	19 (70.37%)	0.175
Totally endoscopic group	36 (44.44%)	8 (29.63%)	
Surgical procedure			0.261
PG	49 (60.49%)	13 (48.15%)	
TG	32 (39.51%)	14 (51.85%)	
Operation time (min)	156.00 (120.00–170.00)	160.00 (120.00–180.00)	0.131
Blood loss (ml)	26.00 (15.00–40.00)	40.00 (15.00–50.00)	0.094

PG, proximal gastrectomy; TG, total gastrectomy.

This study also has some limitations. First, this study is a retrospective study with small sample size. Second, the short postoperative follow-up period was insufficient to collect postoperative prognostic data. Third, the long-term effects of the two different surgical methods cannot be fundamentally evaluated.

In summary, the total laparoscopic transabdominal retromediastinal gastrointestinal reconstruction for Siewert II AEG follows the safety principle of radical tumor resection, has a low economic cost, and can accelerate the recovery of patients in a short time after the operation. It is hoped that future studies will enlarge the sample size and extend the follow-up time to provide more evidence-based medical evidence for the use of fully laparoscopic posterior mediastinal gastrointestinal reconstruction surgery in the treatment of Siewert II AEG patients.

## Data Availability

The original contributions presented in the study are included in the article/Supplementary Material, further inquiries can be directed to the corresponding author/s.
